# Neck‐vein thrombosis during spaceflight

**DOI:** 10.1002/bcp.70300

**Published:** 2025-10-02

**Authors:** Ulrich Limper, Jens Jordan

**Affiliations:** ^1^ German Aerospace Center (DLR) Institute of Aerospace Medicine Cologne Germany; ^2^ Department of Anesthesiology and Intensive Care Medicine, Merheim Medical Center Hospitals of Cologne, University of Witten/Herdecke Cologne Germany; ^3^ Medical Faculty University of Cologne Cologne Germany

**Keywords:** Anticoagulants < Haematology, Cardiovascular < Cardiology, Haemostasis < Haematology, Thrombosis < Haematology

## Abstract

Spaceflight imposes unique environmental challenges, including weightlessness, increased radiation exposure and confinement, which can lead to unexpected health effects. One such example is neck‐vein thrombosis, a condition rarely seen on Earth without predisposing factors such as venous catheters or infections. Although spaceflight has not previously been associated with thromboembolic events, recent cases of neck‐vein thrombosis in astronauts aboard the International Space Station have raised concern. These events were detected incidentally and showed no obvious symptoms, complicating diagnosis. Retrospective studies have not found consistent biomarkers indicating a hypercoagulable state in space, but environmental factors such as weightlessness‐induced venous stasis and potential endothelial damage may contribute. Oral contraceptives could promote thrombotic risk in female astronauts, although no direct link has been confirmed. Research on terrestrial models simulating space conditions has not yet provided conclusive insights into thrombosis prevention. Given the challenges in diagnosing and treating thrombosis in space, agencies such as National Aeronautics and Space Administration and European Space Agency have implemented surveillance programmes and recommended risk‐mitigation strategies, including careful management of venous flow abnormalities and cautious use of anticoagulants. However, more research is needed to refine these strategies and improve safety in future deep space missions.

## 1 INTRODUCTION

Unique environmental conditions in space including weightlessness, increased radiation exposure, isolation and confinement, and altered day–night cycles, can produce unusual and sometimes unexpected health effects. These environmental risks may be exacerbated by preexisting medical conditions and sedentary lifestyle in paying customers who nowadays have access to orbital spaceflight and are less strictly selected and trained than professional astronauts.^1^ A recently discovered medical risk is neck‐vein thrombosis, which is rare in people on Earth^2^ without predisposing risk factors, such as an indwelling venous catheter, trauma, neck infections, or head and neck cancer. The idea that haemostasis could be altered was entertained in the early days of human spaceflight^3^; however, no thromboembolic events had been recognized for more than half of a century.^4^ The exceptionally stressful Apollo 15 lunar mission was associated with serious cardiovascular events in the astronauts in‐ and postflight.^5^ Fifty years later, the first inflight diagnosis^6^ and treatment^7^ of neck‐vein thrombosis has alerted the space medicine community.^8^ This brief review provides an overview on the clinical presentation of neck‐vein thrombosis during spaceflight, potential underlying mechanisms, and preventive and therapeutic pharmacological measures that could be instituted. Because neck‐vein thrombosis can propagate proximally to intracranial veins and sinuses, resulting in stroke,^9^ or embolize into the pulmonary circulation,^10^ detection, prevention and pharmacological treatment are crucial for safe spaceflight. The most prominent symptoms of neck thrombosis, neck pain and headache, may be obscured by the very similar symptoms of space motion sickness^11^ and the puffy‐head syndrome. Therefore, US and European Space agencies recently implemented surveyance programmes to determine the incidence of spaceflight venous thrombosis (SVT) in space.^12^ Furthermore, pharmacological prophylaxis and treatment of SVT pose particular challenges. Weighing potential benefits against bleeding risks in a remote and difficult to access environment with risks for traumatic injuries is complicated by lack of clinical trials data.

## 2 NECK‐VEIN THROMBOSIS IN SPACE

In recent years, cases of thrombosis in the left jugular internal vein of astronauts aboard the International Space Station have been reported.^8^ For instance, a female astronaut in her 40s, with no history of family or personal venous thromboembolism,^13^ developed thrombosis in her left internal jugular vein (IJV) in space.^6^, ^7^ When thrombosis was detected, she was on oral contraceptives to suppress menstruation in space (norethindrone/ethinyl oestradiol).^13^ Except for a prominent left external jugular vein, she was free of specific symptoms, notably headache or worsening of the common facial plethora in space.^7^ Therefore, an occlusive subclinical thrombosis of the internal jugular vein was only recognized as incidental finding during a scientific vascular ultrasound experiment at 50 days in space during a long duration space mission on the International Space Station (ISS).^6^ This astronaut showed a decrease in serum albumin <35 g/L over the flight,^13^ which was retrospectively attributed to oral contraceptive treatment and suspected to have increased thrombotic risk.^13^, ^14^ The astronaut was initially treated with low‐molecular‐weight heparin and subsequently switched to the direct oral anticoagulant apixaban. Antithrombotic treatment was stopped 4 days before return to Earth to reduce bleeding risk during the presumed harsh landing of the Russian Soyuz space capsule.^7^ Interestingly, the thrombus had completely vanished 10 days after landing. No specific cause of IJV thrombosis could be revealed inflight or during postflight thrombophilia work up. Based on the published vascular ultrasound images and the fact vascular compression was not performed, some scientists have questioned the diagnosis of an occlusive thrombus and implicated ultrasound artefact caused by IJV flow disturbances.^15^ Indeed, flow separation and reversed flow, seen for instance after venous stenosis, could also explain the ultrasound findings.^16^, ^17^

Vascular ultrasound data of the same study, which included 11 astronauts, was reviewed retrospectively and a partial thrombus in the left internal jugular vein of a second, male, crew member was suspected.^6^ Furthermore, stagnant or even retrograde flow in the left IJV was seen in 6 of the 11 astronauts.^6^ However, retrospective analyses of plasma samples from 14 astronauts with a mean mission duration of 213 days, including the 2 individuals with IJV thrombosis, did not reveal meaningful alterations in D‐dimer, fibrinogen or anticoagulant biomarkers that would indicate a hypercoagulable state in space.^18^

## 3 EVIDENCE FROM TERRESTRIAL SPACEFLIGHT ANALOGUES

Terrestrial immobilization models such as head‐down tilt bedrest and dry immersion have been extensively applied in healthy, highly selected individuals over the last decades to simulate influences of weightlessness on the human body. Both models have proven useful to delineate physiological influences of weightlessness and to test potential countermeasures for spaceflight. Head‐down tilt bedrest is associated with intravascular volume shifts towards the head, increased cross sectional areas of neck veins,^19^ reduced flow through the right IJV,^20^ but no stagnant or retrograde flow in internal jugular veins have been shown so far. Vein thrombosis, be it in the neck vein or in other vascular beds, has not been described in either model.^21^ However, during short phases of simulated weightlessness on parabolic flight, flow in the IJV reverses or even stagnates.^22^ Those parabolic flight data suggest that bedrest and immersion models do not reproduce all aspects of real weightlessness, such as absence of hydrostatic gradients and tissue compressive forces. Also, additional prothrombotic risk factors, such as stagnant blood flow, may be required during immobilization. Notably, participants in current head‐down tilt bedrest studies undergo broad screening for thrombophilia risk markers and individuals with increased risk are excluded. Terrestrial immobilization models are not yet ready for clinical trials of pharmacological IJV thrombosis prophylaxis or treatment in space.

## 4 MECHANISTIC BASIS

**FIGURE 1:**
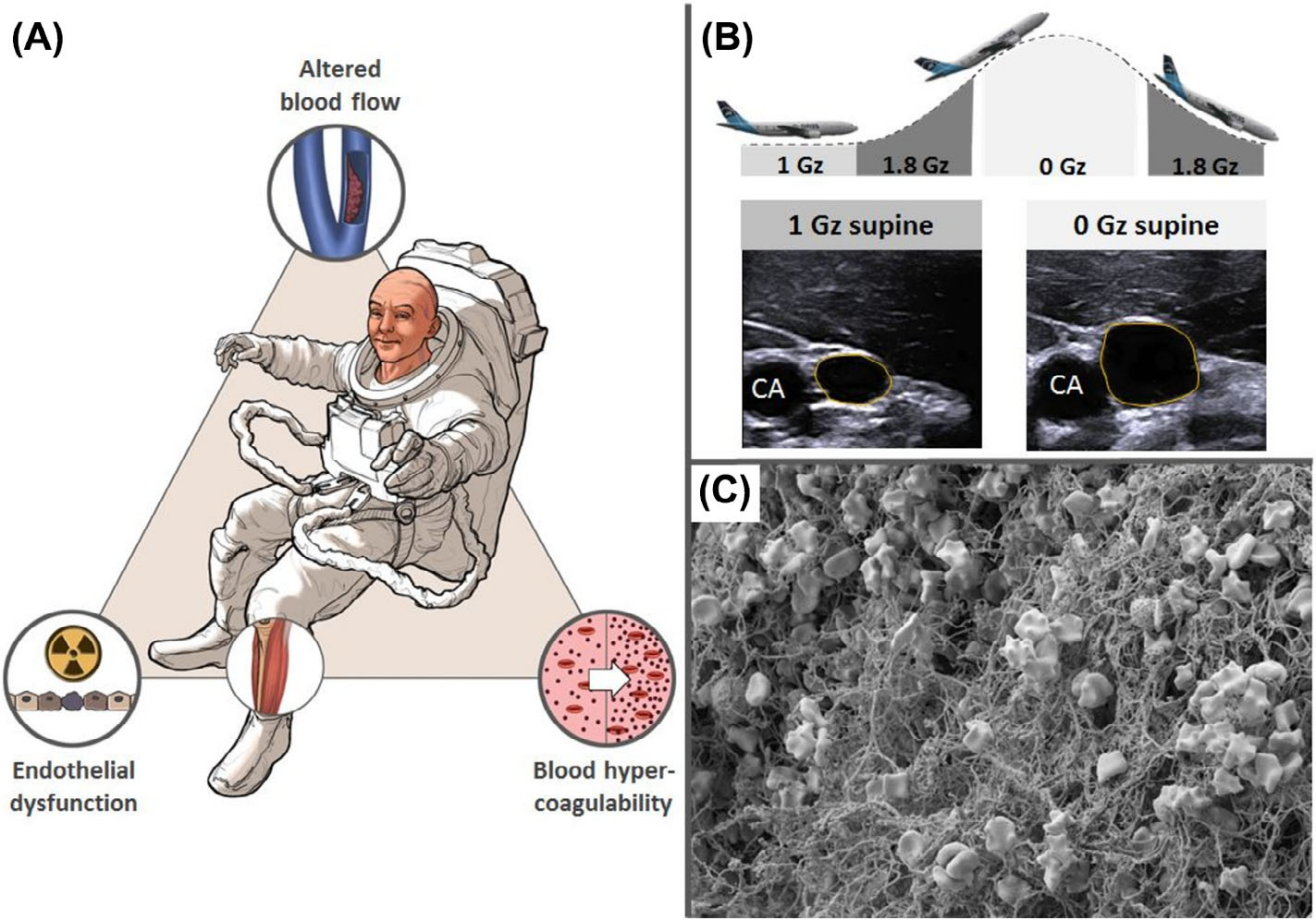
Hypothesized Virchow's triad in space. (A) The environment during spaceflight affects the 3 categories of Virchow's triad stasis, vascular endothelial dysfunction, or hypercoagubility. (B) A representative ultrasound image during weightlessness on parabolic flight shows distension of the internal jugular vein (yellow circle), driven by removal of compressible forces of the surrounding tissues, as the pressure inside is probably lower than it would have been in supine position in 1 Gz. CA, carotid artery; Gz, weight force. (C) Electron microscopy image of a blood clot on earth.

Virchow's triad posits that stasis, vascular endothelial dysfunction or hypercoagubility are required to experience thrombosis (Figure 1). The environment during spaceflight could conceivably affect all 3.^4^, ^23^ More recently, mechanisms protecting from thrombosis during immobilization have been identified.^24^

### 4.1 Stasis

Weightlessness elicits unusual changes in the venous circulation, as the hydrostatic head‐to‐foot gradient is lost and up to approximately 1.9 L of body fluids^25^ and blood are redistributed from legs and abdomen to thorax and head.^26^, ^27^ While venous blood is rapidly shifted towards the head and venous resistance of IJV increase compared to seated position on ground,^6^, ^28^ venous drainage of the head is diverted to rigid vertebral venous plexus when entering weightlessness.^29^, ^30^ Earlier reports on IJV pressure increases in weightlessness^6^, ^28^ are questionable given a flawed measurement technique.^31^ Central venous pressure may actually decrease, remain constant or increase.^27^, ^32^, ^33^, ^34^, ^35^ The phenomenon probably results from differences in volume status, increased whole‐body venous compliance as tissues surrounding veins are unloaded and recruitment of capacitance vessels of the upper body.^34^ Even during prolonged stays in space, reduced venous blood flow velocities, zero flow and sometimes reverse flow may occur, especially in the left IJV where the best documented thrombosis occurred.^6^, ^8^ Structural and flow^30^ asymmetries between both IJVs exist. A study with 190 adult patients in supine position revealed mean cross‐sectional areas of 160 and 102 mm^2^ for the right and left IJV, respectively.^36^

### 4.2 Vascular wall injury

On Earth, prolonged, repeated Valsalva manoeuvres and vascular trauma can increase the risk of experiencing IJV thrombosis.^37^, ^38^ Astronauts have an increased risk for musculoskeletal injuries of the upper body associated with their regular intense inflight resistance exercise protocols or caused by wearing pressurized space suits during extravehicular activities.^39^ The Glenn flight harness^40^ that is worn during exercises in space such as running on the treadmill could also elicit neck vein injury. Finally, other risk factors such as increased radiation exposure in space could also elicit venous endothelial injury via chronic inflammatory processes.^41^

### 4.3 Hypercoagubility

**FIGURE 2:**
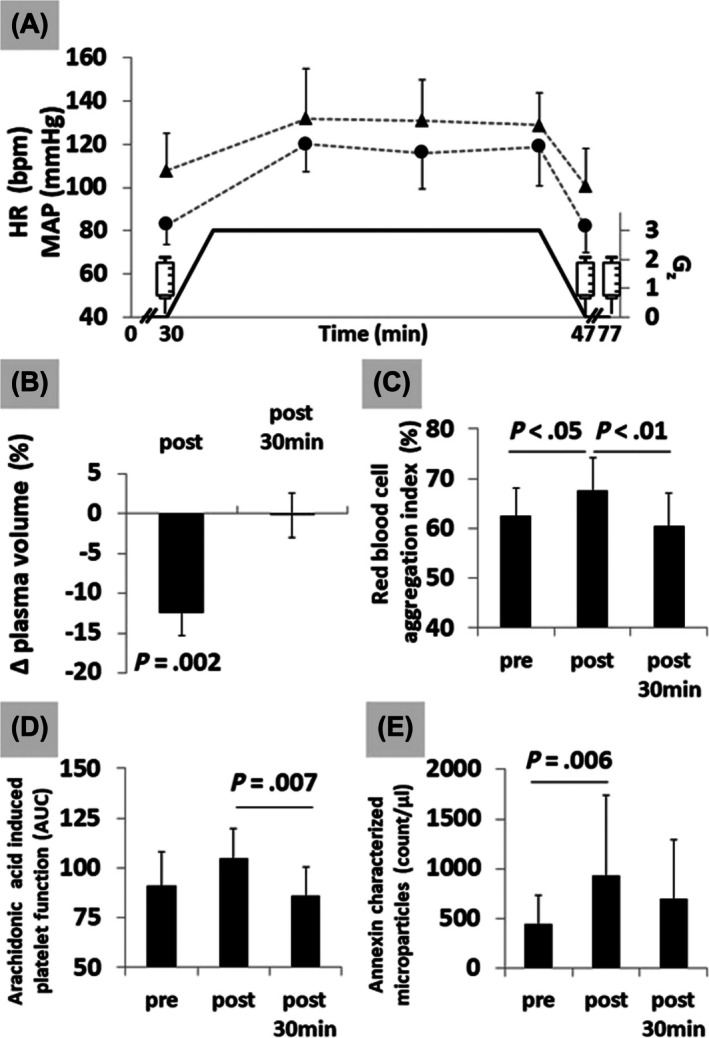
Hypergravity effects on haemostasis. Effects of 15 min of 3 Gz hypergravity on the cardiovascular system, red blood cell function and cell‐based blood coagulation. (A) Hypergravity triggers cardiocirculatory stress responses, heart rate (HR), mean arterial pressure (MAP), syringes indicate time points of blood draw. (B) Relative plasma volume changes indicate significant loss of blood volume. (C) Erythrocyte function is acutely altered by hypergravity. (D) Aggregability of thrombocytes, activated via the arachnoid acid pathway, is increased after hypergravity exposure. (E) Number of thrombocyte‐released microparticles is increased after hypergravity exposure. From Limper U *et al*. Simulated hypergravity activates haemostasis in healthy volunteers. J Am Heart Assoc 2020; 9: e016479. Reprinted with permission from John Wiley and Sons.

Human haemostasis shifts towards a procoagulatory state when the upright human body is exposed to gravity and orthostasis is activated.^42^ Orthostatic hypercoagulability is particularly obvious after the exposure to hypergravity (Figure 2) similar to gravity loads experienced by astronauts during launch and return.^43^

### 4.4 Natural thromboprotection in mammals

**FIGURE 3:**
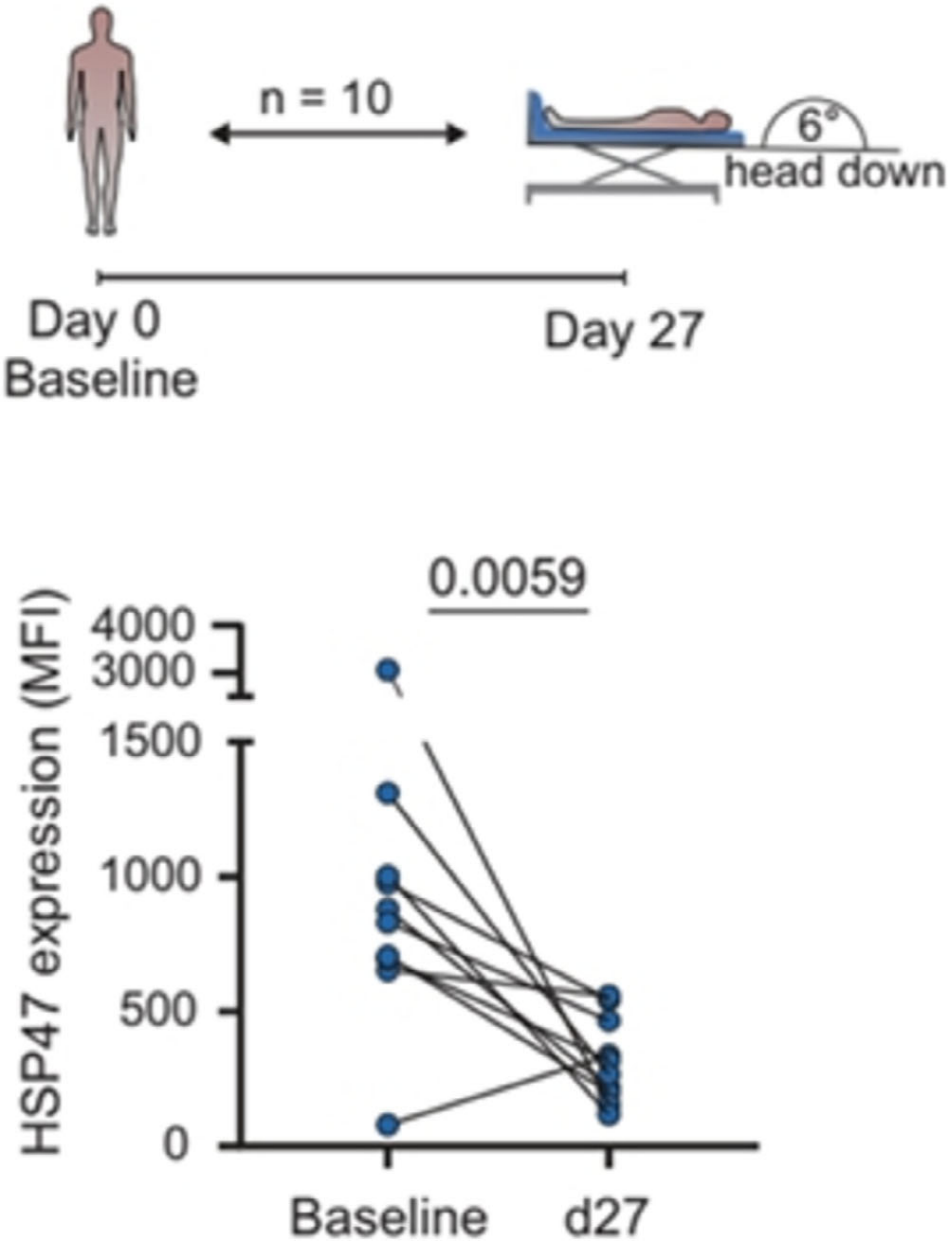
Heat shock protein 47 alterations during bedrest. Natural thrombus protection in 10 healthy bedrest participants by HSP47 downregulation deployed by 27 days of immobilization in −6° degree head town tilt body position. From Thienel M *et al*. Immobility‐associated thromboprotection is conserved across mammalian species from bear to human. Science (New York, NY) 2023; 380: 178–87. Reprinted with permission from AAAS.

Astronauts abide hours to days in the extremely restricted space of a transfer capsule on their way to the ISS without developing any clinical sign of thrombosis, a fact that more than a decade ago has been hypothesized to relate to alterations of immune cells and thrombocytes in microgravity.^44^ Recent investigations identified a potential natural thromboprotection mechanism. Interestingly, hibernating mammals do not suffer from thromboembolic despite several months of immobilization. Patients with spinal cord injury have, after recovery from acute trauma, an almost normal thromboembolic risk. Participants of bedrest studies seem to be protected from thromboembolic events without anticoagulant prophylaxis. In mammals and in humans a mechanism of natural thromboprotection is activated, that works by a reduction of heat shock protein 47, a protein with high importance for activation of platelets and neutrophils and therefore the formation of neutrophile extracellular traps (Figure 3). However, whether the space environment could disable natural thromboprotection deserves to be studied.

### 4.5 Relevance beyond neck veins

These considerations may extend to other medical conditions. Prolonged headward fluid shift, long‐duration space missions may elicit left atrial enlargement, which predisposes to atrial fibrillation and increased thromboembolic risk.^45^ However, many people may be at risk for atrial fibrillation preflight like a commercial astronaut who required pharmacotherapy for frequent ventricular premature complexes to be cleared for spaceflight.^46^ So far, 6 individuals from the National Aeronautics and Space Administration (NASA) astronaut corps have developed atrial fibrillation preflight and flew in space subsequently after therapeutic reestablishment of sinus rhythm.^47^ Thromboembolic risk estimates established on Earth may not be applicable in space.

## 5 RISK MITIGATION IN SPACE

Definite risk factors for spaceflight venous thrombosis have not been identified yet. Each component of Virchow's triad could contribute risk, thus, providing a target for risk mitigation strategies. The European Space Agency and the NASA have recently established guidelines for the prevention, diagnosis and management of spaceflight venous thrombosis to increase general knowledge on venous thrombosis in space and NASA initiated an occupational surveyance programme to evaluate thrombosis in astronauts.^12^ Preliminary results of this programme suggest that venous flow abnormalities cannot solely explain thrombosis in space. Trauma to the upper body, for instance by incorrect worn harness during exercise or activities overloading the musculoskeletal system during extravehicular activities, is thought to promote endothelial dysfunction and inflammation. Hormonal contraceptives are regularly used by female astronauts to suppress menstrual cycle during spaceflight.^48^ Intended effects are less menstrual headache or menorrhagia. Different preparations of hormonal contraception are available, which differ in their ability to increase the risk for venous thromboembolism (VTE), with the highest risk attributed to combined oral contraceptives containing high amounts of oestrogen. However, no VTE in space has been attributed to hormonal contraception. Longitudinal health data from 38 flown female astronauts on combined oral contraceptives did not reveal any sign for VTE.^49^ In contrast, another study investigating a cohort of 65 astronauts, found positive surrogate parameters for increased blood viscosity and increased inflammatory activity in the 13 female astronauts who were on combined oral contraceptives,^13^ theoretically contributing to Virchow's triad. However, it is not known yet whether astronauts should be advised to stop combined oral contraceptives, if further risk factors for VTE, such as venous flow abnormalities, appear in space. NASA recommends continuing oral contraceptives in case of anticoagulation treatment of VTE due to the risk of abnormal uterine bleeding and because any prothrombotic effect can be expected to be suppressed by anticoagulation.^8^

Various thrombophilia markers such as reduced factor C or S or the factor V Leiden mutation identify individuals at increased deep‐vein thrombosis risk. Currently, screening and excluding astronauts for genetic thrombophilia risks would be considered occupational genetic discriminations in many countries. However, such screening could have utility in identifying individuals most likely to benefit from nonpharmacological and pharmacological interventions against neck‐vein thrombosis.

## 6 ANTICOAGULATION IN SPACE—CURRENT DRUGS

Clinical trials of prophylactic antithrombotic or treatment of VTE have not been conducted in space. Due to the low incidence of VTE in astronauts, the limited number of individuals flying into space each year and limited diagnostical and analytical capabilities in space, controlled, randomized, double blind space trials, to test efficacy of antithrombotic drugs appear unrealistic in the foreseeable future. However, astronauts operate in hostile environments. Current missions to the ISS and future manned missions to the moon and Mars are characterized by reduced medical capabilities. Evacuation times from the ISS in case of severe medical incidence are about 1 day. From the Moon, it will take 6–14 days to evacuate and from Mars unplanned evacuation will not be possible.^50^ Therefore, pragmatic treatment approaches in accordance with terrestrial guidelines are recommended. Direct (nonvitamin K) oral anticoagulants, apixaban in particular, have been suggested to be reasonable choices in space.^7^ Administering low molecular weight heparin injections in weightlessness conditions is complex and certified specific antidotes reversal exist for direct oral anticoagulants only (Table 1).^7^, ^72^, ^73^ Vitamin‐K antagonists require regular blood testing and have a narrow therapeutic index classifying them as not optimal for VTE treatment in space. It is important to note that effects of the spaceflight environment on pharmacodynamics and pharmacokinetics have been rarely investigated and studies were limited to analgesics and antimotion sickness medication.^74^ If thrombosis prophylaxis in astronauts is desired, low‐dose apixaban 2.5 mg twice daily may be a rationale choice as this concept has proven successful in preventing deep‐vein thrombosis in cancer patients and after hip replacement.^75^, ^76^ Due to an increased risk for trauma during extravehicular activities and high impact forces during return to Earth, it has been suggested to stop prophylaxis in a timely manner.^77^ Moreover, astronauts are vulnerable to faint and to fall immediately after landing due to postflight orthostatic intolerance.^78^ The first 4 weeks of treatment should be uninterrupted, even if delaying or cancelling an EVA or undocking and return would become necessary.^8^ Treatment of spaceflight venous thrombosis should follow current guidelines on the ground, favouring apixaban due to highest practicability and safety profile.^79^

**TABLE 1:** Overview about different classes of anticoagulants and their applicability in space.

Anticoagulant	Dosing	Therapeutic drug monitoring	Pharmacology	Specific antidotes and reversal agents	Spaceflight applicability for prophylaxis and treatment of VTE
*Oral FXa inhibitors*					
**Apixaban**	• Therapy: 10 mg BID (first 7 days) followed by 5 mg BID (continued therapy) • Prophylaxis: 5 mg (full dose) or 2.5 mg (reduced dose) BID	• Not required • Anti Xa activity or drug level monitoring reasonable in certain cases^51^	CYP3A4‐related risk for drug–drug interaction	• Andexanet alfa^52^ (currently not available on ISS due to high complexity of storage and dose preparation) • 4‐factor prothrombin complex concentrate	Anticoagulant of choice due to low bleeding risk, authorized for prophylaxis and treatment; has been successfully applied in space for treatment of thrombosis
**Rivaroxaban**	• Therapy: 15 mg BID (first 21 days) 20 mg OD (continued) • Prophylaxis: 20 mg or 10 mg OD		CYP3A4‐related risk for drug–drug interaction		Second‐best choice, higher risk for bleeding complications compared to apixaban
**Edoxaban**	• Treatment: 60 mg OD Preceded by LMWH for 5–10 days • Prophylaxis:		P‐gp substrate	• Andexanet alfa (off label) • 4‐factor prothrombin complex concentrate	Initial treatment requires LMWH, therefore no reasonable choice in space due to restricted resources
**Betrixaban**	• Prophylaxis: 80 mg OD		Distinct from other direct oral anticoagulants, due to lowest renal clearance and no CYP metabolism; lower risk for drug interactions.^53^ P‐gp substrate		Marketing authorisation for prophylaxis of DVT only; no marketing authorization in the EU due to increased bleeding risk, thus no reasonable choice
*Oral direct thrombin inhibitors (FIIa)*					
**Dabigatran etexilate**	• Treatment: 150 mg BID Preceded by LMWH for 5–10 days • Prophylaxis: 150 mg BID	Not required. Blood dabigatran concentration; thrombin time^54^ or ecarin clotting time assay^55^ can distinguish subtherapeutic dabigatran levels, currently none of the above are available in space	P‐glycoprotein substrate, Mainly renal clearance, Low risk for CYP‐related drug–drug interaction	Idarucizumab (must be stored refrigerated)	Initial DVT treatment requires LMWH, therefore not a reasonable choice in space due to restricted resources
*Intravenous direct thrombin inhibitors*					
**Bivalirudin (syntetic hirudine)**	• Bolus followed by continuous infusion	aPTT 60–80 s, regular blood draws required	Renal metabolism	Nonspecific, idarucizumab does not work, due to short half‐life (25 min), reversal of action is expected 2–4 h after dose is stopped	Applicable in inpatient care only, primarily in patients undergoing coronary intervention, no indication for application in space
**Argatroban**	• Therapy: continuous infusion of 0.5–2 μg/kg/min	aPTT 60–80 s, regular blood draws required	Hepatically metabolized by CYP3A4^56^	None, due to short half‐life (24 min), reversal of action is expected 2–4 h after stop of infusion	Applicable in inpatient care only
*Oral vitamin K1 recycling antagonists (coumarins)*					
**Warfarin**	Starting dose 5 mg OD; in steady state 2.5 – 10 mg OD	INR 2–3	CYP‐based hepatic metabolism	Vitamin K 4‐factor prothrombin complex concentrate	Due to complex pharmacokinetics no first‐line in space. Point‐of‐care device to measure INR available on ISS.
**Acenocoumarol**	Starting dose 2‐4 mg OD; in steady state 1‐8 mg OD	INR 2–3	Hepatic metabolism		
**Phenprocoumon**	Starting dose 6 mg OD over 2 days, steady state dose 3 mg OD	INR 2–3	Hepatic metabolism, long half‐life of 150 h, treatment takes effect not earlier than 36–72 h after start and continues for 6–7 days after stop, steady state of drug concentration not earlier than 4 weeks after start. Initial bridging with heparin necessary		
*Heparin and LMWHs*					
**Heparin**	• Therapy: continuous intravenous application of 1000 IE/h • Prophylaxis: 7500 IE subcutaneously BID	aPTT 1.5–2.5 times longer than normal, regular blood draws required	Anti FXa/FIIa ratio 1, nonspecific binding to plasma proteins decrease anti Xa and anti IIa response of heparin^57^	Protamine sulfate (1 IE protamine reverses 1 IE heparin). Protamine is available on ISS^58^	Intravenous therapy technically not feasible in space due to regular blood draws and no syringe driver certified for space. Regular monitoring of platelet level necessary for recognizing HIT II. Long‐term therapy is related to osteoporosis, reduced bone density and increased fracture risk^59^
**Enoxaparin**	• Therapy: 1.5 mg/kg OD or 1.0 mg/kg BID (e.g. in patients with pulmonary arterial embolism) • Prophylaxis: 2000 or 4000 IE OD depending on risk level for DVT	• Not required • Measurement of antiFXa activity might be useful in selected cases; however, not available on ISS	Anti FXa/FIIa ratio 3.9, no binding to plasma proteins due to small molecular size^57^	• Andexant alfa (off label)^60^ • Partial reversal by protamine sulfate (1 mg protamine is sought to reverse 1 mg enoxaparin^58^)^61^	Most widely used LMWH on ground.^62^ Available on ISS. Has been successfully used to treat venous thrombosis in space. Risk for HIT II and spontaneous retroperitoneal haematoma.^63^ Better safety profile regarding risk for osteoporosis compared to unfractionated heparin^59^
**Dalteparin**	• Therapy: 200 IE/kg OD or 100 IE/kg BID • Prophylaxis: 2500 or 5000 IE OD depending on risk for DVT		Anti FXa/FIIa ratio 2.5	• 50 mg protamine sulfate reverses 5000 IE of dalteparine in terms of pTT effect and 25–50% of anti FX activity	Risk for HIT II and spontaneous retroperitoneal hematoma.^63^
**Nadroparin**	• Prophylaxis: 3800 or 5700 IE anti Xa OD • Therapy: 4750 to 8550 IE anti Xa BID		• Anti FXa/FIIa ratio 3.5 • Less interaction with platelet function compared to unfractionated heparine^64^	• 6 mg protamine sulfate reverse 950 IE antiXa nadroparine, partial effect of 75% anti‐FXa activity	
**Parnaparin**	• Prophylaxis: 3200–4250 IE aXa OD • Therapy: 6400 or 12 800 IE aXa OD^65^		Anti FXa/FIIa ratio 2.3	• Partial reversal by protamine sulfate	
**Tinzaparin**	• Prophylaxis: 4500 units OD • Therapy: 175 antiXa IE/kg OD		Anti FXa/FIIa ratio 2.0	• Partial reversal by protamine sulfate^61^	
**Certoparin**	• Prophylaxis: 3000 IE OD • Therapy: 8000 IE BID		Anti FXa/FIIa ratio 2.4	• Partial reversal by protamine sulfate (50% of anti‐FXa activity)	
**Bemiparin**	• Prophylaxis: 3500 IE OD		Anti FXa/FIIa ratio 8	• Partial (30%) reversal of anti‐FXa activity by protamine sulfate^66^	
**Reviparin**	• Prophylaxis: 1432 IE OD		Anti FXa/FIIa ratio 4.2	• Partial reversal by protamine sulfate^67^	
*Heparinoids*					
**Fondaparinux**	• Prophylaxis: 2.5 mg OD • Therapy: 7.5 mg OD	• Anti Xa activity	Specific indirect, ATIII mediated, FXa inhibitor	• Protamine is ineffective Andexanet alfa (off label)^68^ •Unspecific stabilization of haemostasis	Increased bleeding risk compared to LMWH,^63^, ^69^ overall little clinical data, therefore no reasonable choice for space
**Danaparoid**	• Prophylaxis (subcutaneous): 750 IE aXa BID • Therapy (intravenous): bolus followed by continuous intravenous infusion	Monitoring of anti Xa activity recommended for renal impairment or overweight	Optimal anticoagulant in case of HIT II as it cleaves heparin‐PF4 complexes.^70^	• None • Unspecific stabilization of haemostasis with fresh frozen plasma	Not authorized for DVT treatment. Treatment of heparin induced thrombocytopenia with and without thrombosis.^71^ Intravenous therapy technically not feasible in space due to regular blood draws and no syringe driver certified for space. Regular monitoring of platelet level necessary

Abbreviations: aPTT, activated partial thromboplastin time; BID, twice daily; CYP, cytochrome P450; DVT, deep‐vein thrombosis; HIT, heparin‐induced thrombocytopenia; INR, international normalized ratio; ISS, International Space Station; LMWH, low‐molecular‐weight heparin; OD, once daily; pTT, partial thromboplastin time; VTE, venous thromboembolism.

## 7 CONCLUSIONS AND CLINICAL OUTLOOK

Neck‐vein thrombosis in space, although rare, presents significant concerns for astronaut health. While the exact causes remain unclear, especially the question of why spaceflight venous thrombosis appears only at the neck, influences of the harsh space environment on venous flow and endothelial damage and hormonal contraceptive use may contribute to thrombotic risk. We speculate that space‐related alterations, limited to the tissues of the neck, may play an important role, such as aberrant blood flow‐driven hypoxaemia or inflammation of vessel walls. The lack of valid biomarkers or conclusive evidence linking these factors to thrombosis complicates diagnosis and prevention. Given the challenges of diagnosing and treating neck‐vein thrombosis in space, future space missions, especially long‐duration flights to the Moon and beyond, will require enhanced surveillance programmes and tailored risk‐mitigation strategies. Pharmacological measures, including anticoagulation therapies, must be carefully evaluated for their safety and efficacy in the space environment, considering the risks of bleeding and trauma. As space exploration continues to advance, further research is needed to understand the full extent of thrombotic risks and develop effective prevention and treatment protocols to ensure astronaut safety on future deep‐space missions.

## AUTHOR CONTRIBUTIONS

**Ulrich** **Limper:** Investigation, writing—original draft. **Jens Jordan:** Investigation and writing—review and editing and supervision.

## CONFLICT OF INTEREST STATEMENT

The authors declare that they have no conflict of interest.

## Data Availability

All data supporting the findings of this report are available within the paper.
